# Transcriptomic analysis of the myometrium during peri-implantation period and luteolysis–the study on the pig model

**DOI:** 10.1007/s10142-014-0401-4

**Published:** 2014-09-21

**Authors:** Anita Franczak, Bartosz Wojciechowicz, Justyna Kolakowska, Kamila Zglejc, Genowefa Kotwica

**Affiliations:** Department of Animal Physiology, Faculty of Biology and Biotechnology, Oczapowski 1A, University of Warmia and Mazury in Olsztyn, 10-718 Olsztyn, Poland

**Keywords:** Myometrium, Transcriptomic profile, Pigs, Pregnancy, Luteolysis

## Abstract

In pigs, implantation begins with the attachment of embryos to the endometrium. As the process is regulated by the expression of numerous genes, endometrial transcriptomic profiles have been extensively studied in early gravid pigs. However, the myometrium, a secretory tissue, should not be neglected, as it can also participate in the regulation of implantation in early pregnant pigs. To clarify this issue, the transcriptomic profile of the porcine myometrium during the peri-implantation period (i.e. on days 15 to 16 of pregnancy) was compared with the profile observed during luteolysis (i.e. on days 15 to 16 of the oestrous cycle) with an Agilent’s Porcine (V2) Two-Colour Gene Expression Microarray 4 × 44 (Agilent, USA). Analysis of the microarray data revealed that of 526 unique, accurately annotated genes, the expression of 271 unique genes was upregulated, while the expression of 255 genes was downregulated in pregnant versus cyclic myometrium. The in-depth data analysis revealed differential expression of genes encoding for factors involved in immunomodulation, tissue growth and differentiation, and prostaglandin and steroid biosynthesis and action. Moreover, the comparison of the obtained data on the myometrial transcriptome with our previously published results on the endometrial transcriptome allowed us to determine substantial differences in the regulatory function of both tissues. The new insights into the function of the myometrium of early pregnant pigs obtained here are in agreement with our previous results that suggest that this tissue plays an important role in providing optimal conditions for developing embryos. Therefore, the importance of the myometrium as an active embryo signal-responsive tissue during early pregnancy cannot be underestimated.

**Electronic supplementary material:**

The online version of this article (doi:10.1007/s10142-014-0401-4) contains supplementary material, which is available to authorized users.

## Introduction

In pigs, the successful maintenance of early pregnancy depends on the secretory activity of the endometrium and the myometrium (Geisert et al. 1982; Franczak and Bogacki 2009). Secretions of these tissues affect the life span of the corpora lutea (CL), thereby leading to continuous progesterone release. Moreover, from the peri-implantation period, from days 13 to 16 of pregnancy, the myometrial contractile activity is limited as a result of various ultrastructural adaptations of this tissue (Thilander and Rodriguez-Martinez 1989). Thus, implantation is not disturbed because of the over-motility of the tissue, and the embryos are therefore protected against deletion.

The present study focussed on alterations in the myometrial transcriptome of early pregnant versus cyclic pigs. In this study, days 15 to 16 of pregnancy and the oestrous cycle were selected as periods of the start of embryo attachment to the endometrium and the onset of luteolysis, respectively (Geisert and Yelich 1997). Previously, we have found that the porcine myometrium synthesises and secretes prostaglandin F_2_ *α* (PGF_2_ *α*) and prostaglandin E_2_ (PGE_2_) (Franczak and Bogacki 2009; Franczak et al. 2004, 2006), while it also produces steroid hormones de novo (Franczak 2008; Franczak and Kotwica 2007, 2010; Franczak et al. 2014; Wojciechowicz et al. 2013). The authors have also documented that the myometrium actively responds to various endocrine factors, e.g. cytokines such as interleukin 1β (IL1β), interleukin 6 (IL6) and tumour necrosis factor α (TNF-α) (Franczak et al. 2010, 2013a, 2014). In pigs, IL1β stimulates myometrial synthesis and secretion of PGF_2_ *α* and PGE_2_ on days 10 to 13 of both pregnancy and the oestrous cycle (Franczak et al. 2010). During the peri-implantation period (on days 15–16 of gestation), IL1β enhances cyclooxygenase 2 messenger RNA (mRNA) expression in the porcine myometrial tissue (Franczak et al. 2010). Our previous study also showed that the cytokines IL1β and IL6, which act during maternal recognition of pregnancy in pigs (on days 12 to 13), increase the release of oestrone (E_1_) by the myometrium in vitro (Franczak et al. 2013a), while IL1β, IL6 and TNF-α stimulate the release of oestradiol 17β (E_2_) on days 15 to 16 of pregnancy (Franczak et al. 2014). Thus, the myometrium was found to be a very intriguing tissue regarding reproductive endocrinology, with a particular function in the regulation of early pregnancy.

The authors are convinced that the myometrium cannot be neglected in the discussion of utero-ovarian interactions occurring both in pregnant and cyclic females. However, the understanding of the endocrine/regulatory role that the myometrium plays during early pregnancy and the oestrous cycle remains limited. Therefore, in this study, transcriptional profiling was performed to identify changes in the myometrium of pigs during the peri-implantation period (days 15 to 16 of pregnancy) compared with luteolysis (days 15 to 16 of the oestrous cycle). The peri-implantation period is critical for the maintenance of pregnancy (Bazer and Thatcher 1977; Bazer et al. 1986; Christenson et al. 1994). We specifically addressed the following questions: (1) how many and exactly which genes are differentially expressed (i.e. upregulated or downregulated) in the porcine myometrium during days 15 to 16 of early pregnancy and days 15 to 16 of the oestrous cycle, (2) what is the interaction network of selected genes encoding important factors responsible for the action of the myometrium that are differentially expressed in pregnant versus cyclic myometria, (3) which biological processes and pathways are significantly altered upon comparison of the transcriptomic profiles of pregnant and cyclic myometria and (4) how many and which genes are differentially expressed in the myometrium (this study) compared with endometrium (Franczak et al. 2013b). To address these questions, the authors utilised whole-genome expression microarrays to elucidate the transcriptional response of the myometrium to the presence of embryos in the uterine lumen.

In this study, domestic pigs were used as a model, as this species is considered to be one of the major mammalian models in biological and biomedical studies because it recapitulates human anatomy and physiology to a large extent. Additionally, many aspects of reproductive function have been studied in the pig, ranging from the basics of maternal-foetal interactions (Green et al. 2006) to embryo development (Sun and Nagai 2003; Gerrits et al. 2005; Rohrer et al. 2006; Tayade et al. 2006). Thus, by using the pig as a model, the authors focussed on reproductive-status-related transcriptomic changes in the myometrium harvested from females during the peri-implantation period and luteolysis. Briefly, our main goal was to identify the transcriptional processes occurring in the myometrium that are potentially associated with the regulation of implantation.

## Materials and methods

### Animals and myometrial tissue collection

Post-pubertal gilts (Large White × Polish Landrace, 90–110 kg) harvested on days 15 to 16 of pregnancy (*n* = 4) or the oestrous cycle (*n* = 4) were used in the experiment. The oestrus behaviour of the gilts was observed in the presence of an intact boar during two consecutive cycles. The onset of the second oestrus was designated as day 0 of the oestrous cycle. Gilts assigned to the early pregnancy group were naturally bred on the second day of oestrus. Pregnancy was confirmed by the presence of embryos after flushing the uterine horns with sterile saline (20 ml). The stage of the oestrous cycle was confirmed by monitoring the morphological changes of the ovaries and CLs (Akins and Morrissette 1968). Immediately after slaughter, the uteri were excised and sections of the middle part of uterine horns were opened longitudinally on the mesometrial surface. The endometrium and the perimetrium were separated from the myometrium by careful scraping using a scalpel blade. Small fragments of the myometrium were then minced, snap frozen in liquid nitrogen and stored at −80 °C. Precision of separation of the myometrium was verified under a dissecting microscope and histologically.

### Ethics statement

All experiments were approved by the Animal Ethics Committee, University of Warmia and Mazury, Olsztyn, Poland.

### RNA isolation and microarray data analysis

RNA isolation, evaluation and the DNA microarray study were performed as described in detail in the previous paper (Franczak et al. 2013b). Briefly, RNA was isolated using a Qiagen RNeasy Mini Kit (Qiagen, Valencia, CA, USA) with DNAse (RNase free DNAse Kit, Qiagen, USA) treatment to digest DNA residues. RNA integrity was evaluated via microfluidic electrophoresis using a 2100 Bioanalyzer (Agilent Technologies, USA). RNA integrity number (RIN) was calculated for each sample using Agilent 2100 Expert software, and samples with an RIN above 8.5 were further processed. The Porcine (V2) Gene Expression Microarrays 4 × 44 (Agilent Technologies, USA) were used. The arrays were processed according to the Two-Colour Microarray-Based Gene Expression Analysis protocol v. 6.6. Total RNA was amplified and labelled with fluorochromes as follows: half of the RNA samples obtained from pregnant animals (*n* = 2) were labelled with Cy3 and the other half (*n* = 2) were labelled with Cy5. The same pattern of labelling was applied to RNA obtained from cyclic animals (*n* = 4) (dye-swap). Labelling was performed using a Low Input Quick Amp Kit (Two-Colour) (Agilent Technologies, USA). After purification of the labelled RNA (Qiagen RNeasy Kit), RNA yield (nanograms of complementary RNA (cRNA)) and specific activity (picomoles of Cy3 or Cy5 per microgram of cRNA) were quantified using an Infinite 200 PRO plate reader with a NanoQuant plate (Tecan Group, Germany). Labelled cRNA was then fragmented, mixed with hybridisation buffer, and placed on the microarray slide. Two differentially labelled cRNA samples (obtained from pregnant and cyclic animals) were placed on each array (*n* = 4) in a balanced block design with dye-swap. The use of four independent biological replicates allowed us to obtain an experiment power of 80 %, with a false discovery rate of 0.1 %, according to the sample size calculation method described by Hu et al. (Hu et al. 2005). The microarrays were then incubated for 17 h at 65 °C in an Agilent hybridisation oven, dissociated from the hybridisation chamber and washed two times in GE wash buffer. After the wash step, the slides were scanned using Agilent’s High-Resolution C Microarray Scanner at the settings recommended for the 4 × 44 K array format. The images obtained after scanning were analysed using Agilent Feature Extraction software v. 10.5.1.1. Analysis included filtering of outlier spots, background subtraction from features and dye normalisation (linear and LOWESS).

### Differentially expressed genes

The data obtained after extraction was further analysed using GeneSpring GX 11.0.2 (Agilent, USA) to determine which genes were differentially expressed in the myometria isolated from pregnant and cyclic pigs. The genes were determined to be differentially expressed if the fold change was greater than 1.2 (upregulation or downregulation). In cases when a statistically altered gene was represented on the Agilent’s Porcine V2 microarray by multiple probes, only the probe set with the largest fold change was reported (the full data set containing multiple probe values for a given gene is presented in Supplementary Table S1.). Probe sets for which both upregulation and downregulation were detected were excluded from further analyses (nine cases). The list of differentially expressed genes was then manually enriched via alignment of the unknown gene probe sequences with the porcine transcriptome using BLAST. Significant differences in gene expression were determined via Student’s *t* test. The differences were considered statically significant at *p* ≤ 0.05.

**Table 1:** Primers used for the validation of microarray results

Gene symbol (official)	Primers sequences	Target sequence accession number
*ANXA1*	Forward: 5′-TTTGATGCTGACGAACTCC-3′ Reverse: 5′-CCAGATGTGTCTGAGGTAATG-3′	NM_001163998.1
*ANXA2*	Forward: 5′-GACGGCTCTGTCATTGATTAT-3′ Reverse: 5′-GACGGCTCTGTCATTGATTAT-3′	NM_001005726.1
*IL13RA2*	Forward: 5′-TCCCTACTTGGAGTCATCAG-3′ Reverse: 5′-GTCTGGTGGCAAAGGTTTA-3′	NM_001243634.1
*HOXA13*	Forward: 5′-ACCTCTGGAAGTCCACTC-3′ Reverse: 5′-CTCCGTTTGTCCTTGGTAAT-3′	NM_001195342.1
*PTGES*	Forward: 5′-TGGTGAGCGGCCAGGTT-3′ Reverse: 5′-TGGCCACTACGTACATCTTGATG-3′	NM_001038631.1
*TLR9*	Forward: 5′-CGAACTCTCAACCTCAAGTG-3′ Reverse: 5′-GGTCGTGATGCTGTTGTAG-3′	NM_213958.1

### Enriched gene ontology terms

The list of differentially expressed genes was uploaded to The *DAVID 6.7*—Database for Annotation, Visualization and Integrated Discovery Classification System (Huang et. al. 2009a; 2009b) to infer the functions of genes based on their evolutionary relationships.

### Interaction network of selected genes

From the population of significantly differentially expressed genes with a fold change ≥1.2, 18 highly altered genes were selected, on the basis of the biological function of the transcript, to construct an interaction network as follows: prostaglandin E receptor, *PTGER3*; microsomal prostaglandin E synthase-1, *PTGES*; steroidogenic acute regulatory protein, *STAR*; annexin A1, *ANXA1*; annexin A2, *ANXA2*; interleukin 13 receptor, alpha 2, *IL13RA2*; homeobox A10, *HOXA10*; homeobox A13, *HOXA13*; interleukin 10 receptor, alpha, *IL10RA*; prostaglandin F receptor, *PTGFR*; androgen receptor, *AR*; Toll-like receptor 9 precursor, *TLR9*; annexin A9, *ANXA9*; vitronectin, *VTN*; cyclin-dependent kinase inhibitor 1B, *CDKN1B*; lectin, galactoside-binding, soluble, 3, *LGALS3*; epithelial cell adhesion molecule, *EPCAM*; and serpin peptidase inhibitor, clade A, *SERPINA7*. The gene interaction network was created with the *GeneMania Prediction Server* (Warde-Farley et al. 2010).

### Comparison of the endometrial and myometrial transcriptomes

The obtained list of genes that were differentially expressed in the myometrium from days 15 to 16 of pregnancy was compared with the list of genes differentially expressed in the endometrium from days 15 to 16 of pregnancy, which was obtained in our previous study (Franczak et al. 2013b). For this purpose, the list of genes differentially expressed in the endometrium was processed using the same methodology as the list of genes altered in the myometrium (this study). The differential expression fold change value cutoff was set to 1.2. For altered genes represented in the microarray by multiple probes, only the probe set with the largest fold change was reported. Ambiguous genes (both upregulation and downregulation reported for one tissue) were excluded from analyses. The comparisons were performed by constructing a Venn diagram for all genes differentially expressed in the myometrium and endometrium as well as for genes solely upregulated and downregulated in the myometrium and endometrium. The diagrams were constructed using the *Venny* online tool (Oliveros 2007).

### Quantitative real-time PCR analysis of gene expression in the myometrium

Six genes (*ANXA1*, *ANXA2*, *IL13RA2*, *HOXA13*, *PTGES* and *TLR9*) were selected for myometrial expression analysis via real-time PCR. Total RNA samples (*n* = 4 for the implantation period and *n* = 4 for luteolysis) were transcribed into cDNA using an Omniscript RT Kit (Qiagen), dNTPs and random hexamers as primers. Real-time PCR was performed using a 7300 Real-Time PCR System and SYBR® Green PCR Master Mix (both Life Technologies, Grand Island, NY, USA). The initial denaturation was carried out at 95 °C for 10 min, followed by 40 cycles of denaturation at 95 °C (15 s) and primer annealing and elongation at 60 °C (60 s). All amplifications were followed by dissociation curve analysis of the amplified products. Non-template controls were used for each set of primers to confirm reaction specificity. The specificity of amplifications was further confirmed via electrophoresis of the PCR products on a 2 % agarose gel. Specific primers (Table 1) were designed using the Primer Express 3.0 software (Life Technologies), and primer specificities were confirmed with Basic Local Alignment Search Tool (BLAST). Gene expression levels were calculated using the ΔΔCt method and normalised using the geometric mean of the expression levels of two reference genes—glyceraldehyde 3-phosphate dehydrogenase (*GAPDH*; Bogacka et al. 2006) and β-actin (*ACTB*; Staszkiewicz et al. 2007). The significant difference in gene expression between the myometrium of pigs during the implantation and luteolytic periods was analysed via Student’s *t* test. Confirmed differences in gene expression were expressed as fold changes.

## Results

### Description of differentially regulated genes

The significant, pregnancy-induced change in expression of 526 genes (unique and accurately annotated in GeneBank) in the myometrium obtained from pregnant pigs (days 15 to 16) compared with the myometrium obtained from cyclic pigs (days 15 to 16) was detected (Supplementary Table S1). Out of these 526 genes, 271 genes were statistically upregulated and 255 genes were downregulated. Among the significantly differentially expressed genes, the expression of 70 unique transcripts displayed more than a twofold change (39 upregulated and 31 downregulated). The top 20 upregulated and downregulated genes in the myometrium are presented in Supplementary Table S2. Certain genes encoding known important factors involved in the regulation of the myometrium, including endocrine activity and myocyte differentiation, were differentially expressed in the tissue harvested from pigs during the peri-implantation versus luteolysis period. The significantly upregulated genes included the following: *HOXA13* (homeobox A13; 2.54, *p* = 0.05), *PTGER3* (prostaglandin E receptor 3; 2.40, *p* = 0.038), *ANXA2* (annexin A2; 1.77, *p* = 0.008), *HOXA10* (homeobox A10; 1.61 *p* = 0.04), *STAR* (steroidogenic acute regulatory protein; 1.60, *p* = 0.05), *ANXA1* (annexin A1; 1.53, *p* = 0.03), *IL10RA* (interleukin 10 receptor, alpha; 1.31, *p* = 0.008), *PTGES* (microsomal prostaglandin E synthase-1; 1.22, *p* = 0.04) and *IL13RA2* (interleukin 13 receptor, alpha 2; 1.22, *p* = 0.03). The downregulated genes included the following: *PTGFR* (prostaglandin F receptor; −1.29, *p* = 0.009), *TLR9* (Toll-like receptor 9 Precursor; −1.22, *p* = 0.04) and *AR* (androgen receptor; −1.20, *p* = 0.03).

**Table 2:** The list of significantly enriched GO terms

Gene list	GO category	GO term	Description	*p* value	Gene count/percent
Upregulated in the myometrium from pregnant gilts (days 15 to 16)	GOTERM_BP_FAT	GO:0002474	Antigen processing and presentation of peptide antigen via MHC class I	1,99E-02	3/2.29
	GOTERM_BP_FAT	GO:0048002	Antigen processing and presentation of peptide antigen	1,99E-02	3/2.29
	GOTERM_CC_FAT	GO:0048770	Pigment granule	1,45E-02	5/3.81
	GOTERM_CC_FAT	GO:0042470	Melanosome	1,45E-02	5/3.81
	GOTERM_CC_FAT	GO:0044421	Extracellular region part	6,54E-02	11/8.39
	GOTERM_CC_FAT	GO:0031410	Cytoplasmic vesicle	6,87E-02	6/4.58
	GOTERM_CC_FAT	GO:0031982	Vesicle	6,87E-02	6/4.58
	GOTERM_MF_FAT	GO:0043169	Cation binding	2,58E-02	28/21.3
	GOTERM_MF_FAT	GO:0043167	Ion binding	2,81E-02	28/21.3
	GOTERM_MF_FAT	GO:0046872	Metal ion binding	6,32E-02	26/19.8
Downregulated in the myometrium from pregnant gilts (days 15 to 16)	GOTERM_BP_FAT	GO:0007218	Neuropeptide signaling pathway	5,81E-03	7/5.51
	GOTERM_CC_FAT	GO:0005840	Ribosome	4,09E-03	7/5.51
	GOTERM_CC_FAT	GO:0030529	Ribonucleoprotein complex	9,06E-03	7/5.51
	GOTERM_MF_FAT	GO:0005198	Structural molecule activity	5,16E-03	10/7.87
	GOTERM_MF_FAT	GO:0003735	Structural constituent of ribosome	5,90E-03	7/5.51
	GOTERM_MF_FAT	GO:0003707	Steroid hormone receptor activity	5,71E-02	4/3.14
	GOTERM_MF_FAT	GO:0004879	Ligand-dependent nuclear receptor activity	6,41E-02	4/3.14

*GO* gene ontology

### Enriched gene ontology terms

The assignment of gene ontology (GO) terms to the lists of upregulated and downregulated genes revealed a significant enrichment of ontologies associated with the following GO terms: antigen processing and presentation (GO:0002474 and GO:0048002), cytoplasmic vesicle (GO:0031410), vesicle (GO:0031982), cation, ion and metal ion binding (GO:0043169, GO:0043167 and GO:0046872, respectively), neuropeptide signalling pathway (GO:0007218), ribosome (GO:0005840), ribonucleoprotein complex (GO:0030529), structural molecule activity (GO:0005198), structural constituent of ribosome (GO:0003735) and steroid hormone receptor activity (GO:0003707), among others. The full list of significantly enriched gene ontologies is reported in Table 2.

### Interaction network of selected genes

**Fig 1:**
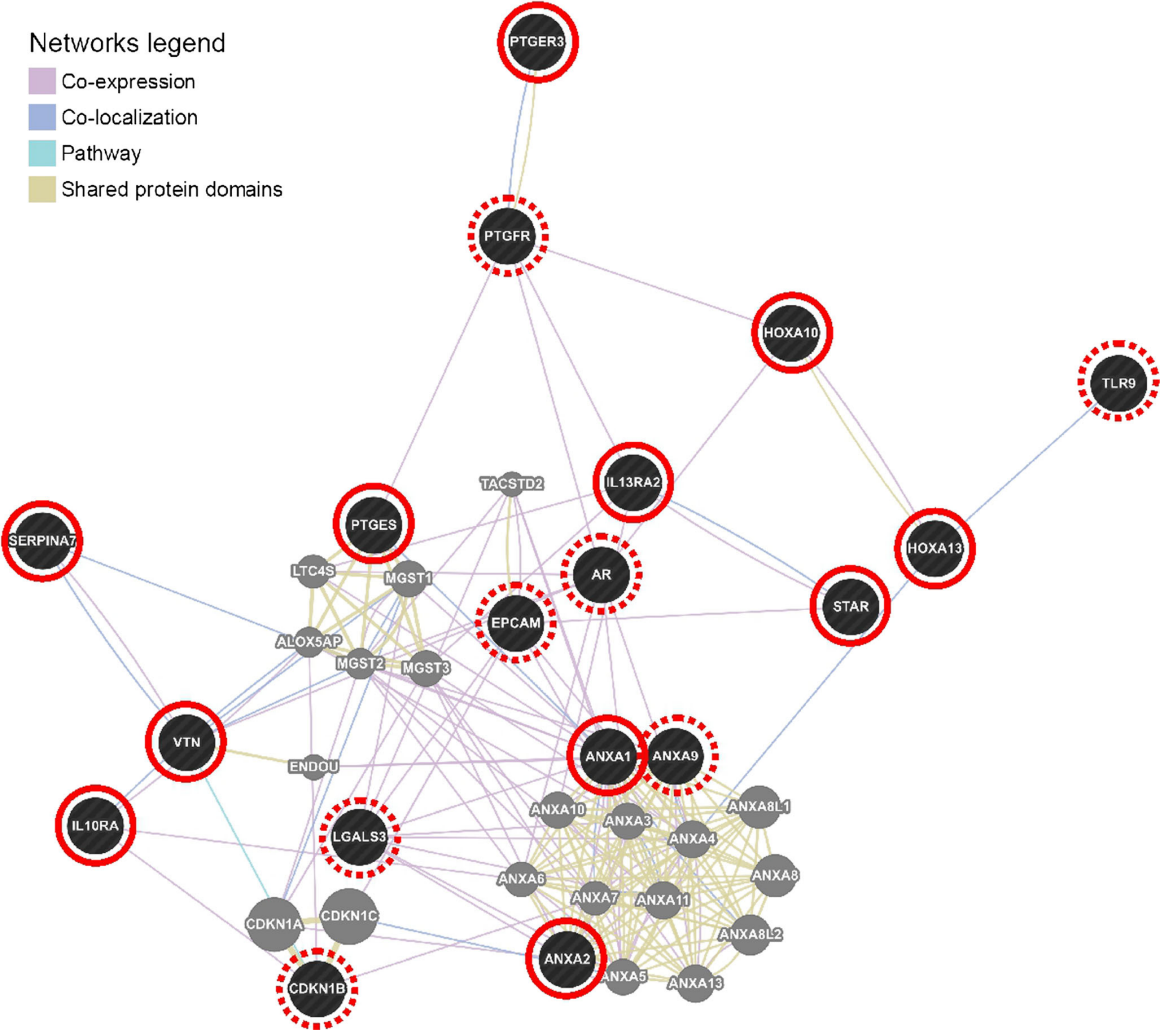
Gene interaction network constructed with GeneMania for 18 highly altered genes. The *colour* of the line that connects the genes depicts the type of interaction (see *legend*). The *solid red circles* indicate upregulated genes, while the *dashed circles* indicate downregulated gene

In the constructed regulatory network (Fig. 1), all 18 query genes were connected. The majority of significant interactions were co-expression-based (78 interactions) and co-localisation-based (19 interactions).

### Comparison of the endometrial and myometrial transcriptomes

**Fig 2:**
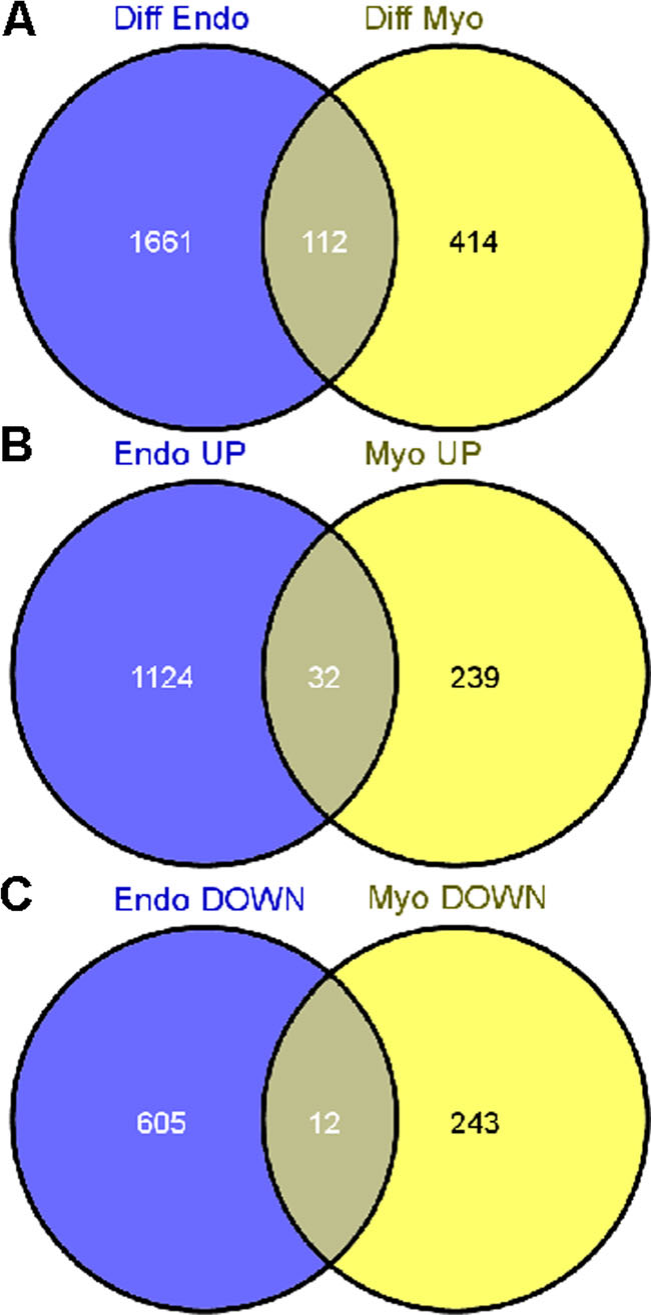
Venn diagrams representing genes commonly differentially expressed in the myometrium (*Myo*; this study) and the endometrium (*Endo*; Franczak et al. 2013b) from days 15 to 16 of pregnancy. **a** Comparison of all differentially expressed genes; **b** comparison of upregulated genes; **c** comparison of downregulated genes

Among the differentially expressed genes (1,773 genes in the endometrium and 526 genes in the myometrium), the expression of 112 genes were commonly altered; 32 genes were upregulated and 12 genes were downregulated both in the endometrium and the myometrium from days 15 to 16 of the pregnancy (Fig. 2). Sixty-eight genes were differentially regulated in the endometrium and myometrium (i.e. upregulated in the endometrium and downregulated in the myometrium or downregulated in the endometrium and upregulated in the myometrium). The *AR* gene encoding for androgenic receptor was downregulated in both tissues, while the *PTGES* gene encoding microsomal prostaglandin E synthase was upregulated in the myometrium and downregulated in the endometrium isolated during the peri-implantation versus luteolysis periods.

### Validation of the DNA microarray data for the myometrium

To validate the DNA microarray data, six genes were selected. The genes selected for real-time PCR (Table 3) are involved in the following aspects of the regulation of early pregnancy: PGs synthesis (*PTGES)*, uterine development and remodelling (*HOXA13*), innate immune response (*TLR9*) and interleukin 13 response (*IL13RA2*), immunomodulation, anticoagulant function and anti-inflammatory actions of glucocorticoids in the host defence system (*ANXA1* and *ANXA2*). The data obtained with the microarrays were confirmed via real-time PCR (Table 3).

**Table 3:** Results of selected gene expression validation with real-time PCR

Gene symbol	Microarray fold-change	Real-time PCR fold change	Regulation during pregnancy
*ANXA1*	1.53	2.12	Up
*ANXA2*	1.51	1.82	Up
*IL13RA2*	1.22	1.11	Up
*HOXA13*	2.54	2.16	Up
*PTGES*	1.22	1.73	Up
*TLR9*	−1.22	−1.24	Down

## Discussion

In the current study, the Agilent DNA microarray technique was used to determine and compare gene expression patterns in the myometrium of pigs during the peri-implantation period (days 15 to 16 of pregnancy) and luteolysis (days 15 to 16 of the oestrous cycle). We provide a complete list of annotated genes that are differentially expressed in the myometrium isolated from females during the peri-implantation period compared with those expressed in the myometrium during the period of luteolysis. The novel aspect of this study is that the two transcriptomes (i.e. determined in pregnant and cyclic pigs) were compared to identify the known genes that are uniquely expressed in the myometrium harvested from pregnant females.

In this study, we determined that 526 accurately annotated, unique genes were differentially expressed in the peri-implantative versus luteolytic myometrium. From these genes, 271 genes were upregulated and 255 were downregulated. Interestingly, among the differentially expressed genes in the porcine myometrium, transcripts encoding factors responsible for steroid and prostaglandin synthesis and action, uterine development and remodelling as well as innate immune response and growth factor production were found. These observations again confirm the substantial role that the myometrium plays in the maintenance of early pregnancy in pigs not only in shape retention and contracting but also as tissue involved in endocrine and immune signalling.

Until now, the role that the myometrium plays in implantation and CL protection in early gravid pigs and regulation of luteolysis in cyclic pigs has been almost completely neglected. We have previously demonstrated that on days 15 to 16 of pregnancy and the oestrous cycle in pigs, cytokine action, steroidogenesis and prostaglandin synthesis take place in the myometrial tissue (Franczak 2008; Franczak and Kotwica 2007, 2010; Franczak et al. 2004, 2006, 2010, 2013a, 2014). The present results have confirmed that alterations in the expression of genes responsible for immune response, PGE_2_ synthesis (*PTGES*), PGE_2_ (*PTGER3*) and PGF2α (*PTGFR*) response, and cell adhesion and steroid hormone response also occur in the myometrial transcriptome. Interestingly, our results demonstrate that *PTGES* and *PTGER3* are upregulated in the early pregnant myometrium. This notion again confirms our suggestion that the myometrium may be an important source of luteotrophic PGE_2_ in pregnant pigs and that it is more suitable for the response to PGE_2_ production in pregnant than cyclic pigs (Franczak et al. 2006, 2010; Franczak and Bogacki 2009).

We found that in the early pregnant porcine myometrium, *PTGFR*, which encodes receptor for luteolytic PGF_2_α, was among the downregulated genes. The decreased expression of *PTGFR* indicates the reduced ability of the myometrium to respond to PGF_2_α. Therefore, we suggest that this phenomenon is very important for successful implantation. It is important to note that in addition to luteolytic action, PGF_2_α may act also as contracting, proinflammatory and immunostimulatory factor (Lewis 2004; Langendijk et al. 2002). Thus, the phenomenon of *PTGFR* mRNA downregulation in the myometrium enables the onset of implantation and may mediate the protection of developing embryos from mobilisation of immune cells in the pregnant uterus.

The existence of an immunosuppressive mechanism directly in the porcine myometrium was further supported by the decreased expression of *TLR9* in this tissue*.* Toll-like receptors (TLRs) are involved in the activation of the innate immune system via induction of inflammatory cytokines or interferons (Fazeli et al. 2005; Takeda et al. 2003). It is important to note that the decreased expression of *TLR9* and *PTGFR* was not observed in the early pregnant endometrium of pigs (Franczak et al. 2013b). Thus, the myometrial tissue in pregnant gilts may be responsible for the protection of the embryo against proinflammatory and immunostimulatory signals mediated via PGF_2_α or activation of the innate immune system. The phenomenon of *TLR9* mRNA decreased expression in the porcine myometrium during onset of implantation is especially interesting. It was found that TLR9 activation coupled with IL10 deficiency in mice induces adverse pregnancy complications (Goulopoulou et al. 2012). Thus, the decreased expression of TLR9 mRNA in the myometrium may protect against pregnancy complications in pigs.

In this study, the upregulation of genes encoding for the IL10 and IL13 receptors was found in the porcine myometrium. Previously, it was determined that IL10 and IL13 are among the most important cytokines for a successful pregnancy (Rivera et al. 1998; Itoh et al. 2007). The cytokines promote growth and counteract cytokines that are deleterious for early pregnancy (Maj and Chelmonska-Soyta 2007; Viganò et al. 2001). IL13 can stimulate growth factor production (Itoh et al. 2007). Thus, the upregulated receptors of both cytokines in the porcine myometrium may contribute to successful implantation in pigs.

In pregnant pigs, we also observed the upregulation of genes encoding homeobox A family proteins (HOXA10 and HOXA13), which are known to affect uterine development and remodelling, the processes required for successful implantation and placentation (Ekici et al. 2013; Shaut et al. 2008; Zhao and Potter 2001). In the early pregnant porcine myometrium, these proteins regulate tissue rebuilding, thereby adapting the tissue to the developing pregnancy (Thilander and Rodriguez-Martinez 1989). We suggest that the activity of HOXA10 and HOXA13 may contribute to the differentiation and growth of myocytes during the peri-implantation period in pigs and may be involved in the morphological and ultrastructural adaptation of the myometrium to pregnancy (Thilander and Rodriguez-Martinez 1989).

In this study, the androgen receptor encoding gene (*AR*) was significantly downregulated in the early pregnant myometrium compared with the myometrium during luteolysis*.* In pigs, the primary circulating androgen is androstenedione (A_4_), which serves as the principal substrate for E_1_ synthesis (Simpson et al. 2001). Moreover, an increased supply of androgens A_4_ and testosterone (T) to the uterus was determined in pigs during the maternal recognition of pregnancy and embryo implantation (Stefanczyk-Krzymowska et al. 1998). Androgens are also synthesised and secreted locally by the porcine myometrium during both early pregnancy and luteolysis (Franczak 2008; Franczak and Kotwica 2010). Androgen receptors are present in the porcine uterine tissues during early pregnancy (Cárdenas and Pope 2003). Interestingly, androgens in pigs may decrease blastocyst survival (Cárdenas et al. 2002) and downregulate oestrogen receptors in the myometrium (Cárdenas and Pope 2004). Taken together, we suggest that the decreased expression of *AR* may protect both the myometrium (the present study) and the endometrium (Franczak et al. 2013b) against negative androgen action during the peri-implantation period in pigs. These observations are further supported by the downregulation of steroid hormone receptor activity ontology (GO:0003707) in the early pregnant myometrium, as observed in the current study. Overall, the precise control of steroid action in uterine tissues seems to be crucial for the maintenance of pregnancy during the pre- and peri-implantation period.

In the current study, we found the differential expression of certain genes encoding factors potentially important for the regulation of myometrial endocrine functions—annexin 1 (ANXA1) and 2 (ANXA2). Genes encoding for both annexins were significantly upregulated in the early pregnant myometrium. It has been previously determined that ANXA1 and ANXA2 are both structurally and functionally similar (Liemann and Huber 1997). Both molecules possess strong immunosuppressive and anti-inflammatory properties (Aarli and Matre 1998) and are potent endocrine regulators (Gerke and Moss 2002). The expression of *ANXA1* has been determined in reproduction-associated organs with endocrine activity such as the ovary (Tsao et al. 1995), placenta (Fava et al. 1989) and myometrium (current study), but not in the endometrium at the peri-implantation stage of pregnancy in pigs (Franczak et al. 2013b). It was found also that ANXA1 reduces the in vitro production of testicular T (Cover et al. 2002); therefore, similar impact on the previously described myometrial androgen release cannot be excluded (Franczak 2008; Franczak and Kotwica 2010). The inhibition of androgen synthesis together with the downregulation of *AR* and downregulation of steroid hormone receptor activity ontology, discussed above, may protect the implanting embryos from over-exposition to androgenic activity.

Interestingly, the expression of *ANXA1* is stimulated by E_2_ (Castro-Caldas et al. 2001), the most important signal for maternal recognition of pregnancy in pigs (Bazer and Thatcher 1977). It is known that E_2_ can be synthesised not only by embryos but also locally in the porcine endometrium and myometrium (Franczak and Kotwica 2007, 2010; Franczak et al. 2014). Endometrial and myometrial E_2_ may participate in the regulation of ANXA1 expression in an autocrine and paracrine manner. This concept, once again, confirms that this hormone plays an important role in the establishment and maintenance of pregnancy in pigs, not only as a signal at the embryo-maternal interface but also as a potent and multidirectional regulator of uterine activity.

Alterations in the transcriptomic profiles on the same days (15 to 16 of pregnancy or the oestrous cycle) as assessed in this study were established previously in the porcine endometrium (Franczak et al. 2013b). After adjusting these results, according to the method used in this study, 1,773 genes were differentially expressed (more than 1.2-fold); 1,156 genes were upregulated and 617 genes were downregulated in the early pregnant versus cyclic endometrium (Franczak et al. 2013b; data adjusted). Thus, we observed a 3.4-fold lower gene change during the peri-implantation period in the porcine myometrium compared with the endometrium, thereby indicating a different contribution of these tissues in the maintenance of implantation. As embryos start to contact the endometrium on days 15 to 16 of pregnancy, the more abundant changes in the endometrial versus myometrial transcriptome seem to be fully legitimate. Thus, the current study indicates that during the peri-implantation period in pigs, the transcriptome of the myometrium is more stable than the transcriptome of the endometrium.

This study provides, for the first time, a complete list of upregulated and downregulated genes in the early pregnant porcine myometrium. In summary, these data will help to define the complex patterns of myometrial genes acting to create the environment required for implantation and development of porcine embryos. Generally, the data indicate that, on the transcriptomic level, the porcine myometrium and endometrium respond differently during the peri-implantation period. Because the pig can be utilised as a model for biomedical studies and because some of the mechanisms described above also reflect human physiology, the results may guide the future research of physiology and pathophysiology of early pregnancy.

## Electronic supplementary material

Below is the link to the electronic supplementary material.

ESM 1 (PDF 493 kb)

ESM 2 (PDF 27 kb)
