# Supplementary material for: Transcriptomic analysis of the myometrium during peri-implantation period and luteolysis–the study on the pig model
Source: Funct Integr Genomics. 2014 Sep 21;14(4):673–82. doi: 10.1007/s10142-014-0401-4 (PMC4233113; doi:10.1007/s10142-014-0401-4)
Supplement: Supplementary file 1 — (PDF 493 kb) [file 10142_2014_401_MOESM1_ESM.pdf]

Article title: Transcriptomic analyses of the myometrium during periimplantation period and luteolysis - the study on the pig model

Journal name: Functional & Integrative Genomics

Author names: Anita Franczak*, Bartosz Wojciechowicz, Justyna Kolakowska, Kamila Zglejc, Genowefa Kotwica

Affiliation: Department of Animal Physiology, Faculty of Biology and Biotechnology, Oczapowski 1A, University of Warmia and Mazury in Olsztyn, 10-718 Olsztyn, Poland

*Corresponding author: phone: +48 89 5233201; fax: +48 89 5233937
E-mail address: anitaf@uwm.edu.pl (Anita Franczak)

Gene symbol	fold-change	direction	p-value
AASS	1.1662343	up	0.016237715
ACSL3	1.5508583	up	0.006565776
ACSL3	1.5540037	up	0.023707263
ACSL4	1.3756901	up	0.044231787
ACTA1	1.3072255	up	0.0149981
ACTA1	1.3175708	up	0.048888054
ACTA1	1.3505844	up	0.028769674
ACTA2	1.350696	up	0.009649821
ACTB	1.4152246	up	0.00008146511
ADAL	1.52726	up	0.0071253376
ADAMTS1	1.5729971	up	0.04289601
ADD3	1.2269548	up	0.025714902
ADRA2B	1.6516705	up	0.044579674
AFP	1.6643608	up	0.038138166
AGPAT6	1.4253705	up	0.03156776
AKAP9	1.5866001	up	0.026440607
ALB	1.1997147	up	0.0019535937
AMCF-II	1.4338427	up	0.0055857603
AMY2	1.1299549	up	0.043191
AMY2	1.1507771	up	0.0065235114
ANKRD17	1.1920246	up	0.008036961
ANKS1A	1.319347	up	0.035890557
ANXA1	1.3848825	up	0.01918302
ANXA1	1.3925256	up	0.012141592
ANXA1	1.5326735	up	0.02788961
ANXA2	1.5151542	up	0.008236823
ANXA2	1.7748194	up	0.008485597
APPBP2	1.2764686	up	0.019771008
ARHGAP31	1.6059262	up	0.025580784
ARL4C	1.5397075	up	0.049566533
ARL4C	1.8368278	up	0.046720285
ARMC9	2.6355546	up	0.03473136

ASPN	2.4304504	up	0.0039201975
ATE1	1.3457229	up	0.01616976
ATF4	1.294333	up	0.010076752
ATP1A1	1.2145725	up	0.029537551
ATP2C1	1.1737995	up	0.031833444
ATP2C1	1.5848913	up	0.01065256
ATP6V1D	1.2983087	up	0.011957047
ATP8B4	2.206954	up	0.02892049
ATRIP	1.6082098	up	0.022791067
BLCAP	1.1674255	up	0.030033974
BMP1	1.2235364	up	0.014767843
BZW1	1.4165229	up	0.030206848
C1QTNF7	1.501588	up	0.049772743
CA3	2.101705	up	0.013715045
CA3	2.6526773	up	0.011317536
CAK1	1.1700121	up	0.03752837
CALM2	1.4047202	up	0.0036684512
CALM3	1.3222032	up	0.0013157072
CAMKK2	1.531001	up	0.0008236741
CAPZB	1.1738003	up	0.04546916
CAST	1.2374138	up	0.032244913
CATSPERB	1.4165981	up	0.030110586
CAV1	2.1266959	up	0.022373503
CC2D2A	1.4856282	up	0.0047702584
CCDC88A	1.2619011	up	0.0049046027
CCR1	3.492307	up	0.0009701039
CCR3	3.492307	up	0.0009701039
CCXCR1	3.492307	up	0.0009701039
CD163L1	1.3444725	up	0.049309567
CDC42BPB	1.6951385	up	0.04185372
CLCC1	1.4730536	up	0.039253194
CLIP1	1.3979797	up	0.0466256
COL21A1	1.2837429	up	0.00028626487
COL21A1	1.4231853	up	0.029785309
COLCA2	1.7983773	up	0.028244449
COLL11A1	1.7426089	up	0.023562575
COPA	1.3749311	up	0.028925568
CPEB1	1.3242329	up	0.027995413
CRSP-2	1.295813	up	0.02525998
CTHRC1	1.4205631	up	0.002352825
CTSB	1.6936924	up	0.049343757
CYB5A	1.3464575	up	0.043773055
CYCS	1.9830613	up	0.02028209
CYP27A1	1.3372605	up	0.009674533
DCAF8	1.098654	up	0.032391023
DCAF8	1.2822034	up	0.04293986

DDX18	1.3583202	up	0.030475633
DEK	1.2038716	up	0.04811694
DHCR24	1.2264462	up	0.037717838
DMBT1	2.0010815	up	0.004736472
DMD	1.3578068	up	0.026958406
DTD1	1.2397443	up	0.0041894717
DYNC1LI2	1.5165192	up	0.026413145
DYNC1LI2	1.8388641	up	0.04434123
EIF3E	1.32937	up	0.021667663
ENO3	2.2956975	up	0.019150672
ENPP1	4.5354414	up	0.023956046
EPS15L1	1.5295978	up	0.043948438
ETV3	4.6618896	up	0.033025183
FAM63B	2.0869927	up	0.042273186
FAM69A	1.2289655	up	0.0020950797
FETUIN	1.5615293	up	0.028421076
FHL1C	1.1447881	up	0.026505183
FHL1C	1.3446567	up	0.013294668
FHL1C	1.3672885	up	0.0011494071
FHL1C	1.3761892	up	0.00003433124
FHL1C	1.5224366	up	0.0029632405
FIL1L	1.7210178	up	0.03115328
FIL1L	1.9434379	up	0.046273947
FIL1L	2.3357441	up	0.036851913
FKBP3	1.260536	up	0.04989614
FLOT1	1.7397768	up	0.04009709
FOXN2	1.4924011	up	0.016432175
FOXP1	1.6550816	up	0.013747984
FYB	2.2846353	up	0.0021516823
FYCO1	3.492307	up	0.0009701039
GAB3	1.1459491	up	0.0076499376
GALNT7	1.6271416	up	0.026069412
GBF1	1.6606405	up	0.040455833
GLT25D1	1.4214336	up	0.039890815
GNAI2	1.3154764	up	0.038538214
GNAI2	1.3923675	up	0.035930324
GOLGA3	1.3901141	up	0.04813427
GOLGA4	1.3399612	up	0.014420964
GOLGA4	1.5990752	up	0.022849686
GPM6B	2.3103452	up	0.032330263
GRB10	1.6945372	up	0.0075397613
GSN	2.3015935	up	0.044253472
HDDC2	1.6544315	up	0.009211044
HDLBP	1.8592265	up	0.029153477
HEXB	1.2261558	up	0.03090165
HEXB	1.2382355	up	0.0012689751

HEXB	1.288688	up	0.0055792485
HEXB	1.470827	up	0.025260208
HGFR	2.7961392	up	0.015068111
HIAT1	1.5952559	up	0.016208
HJURP	1.4415303	up	0.003086891
HNRNPAB	1.4490271	up	0.032149125
HOXA10	1.6109545	up	0.04347795
HOXA13	2.5405235	up	0.048123196
HSDM	1.2537476	up	0.013381558
HSP70.2	2.014736	up	0.018915497
HSP90B1	1.091137	up	0.048553247
HSP90B1	1.4345274	up	0.044780057
HTC	1.0686175	up	0.019779216
HTC	1.2293679	up	0.027327925
IARS2	3.0902135	up	0.01084113
ICK	1.3296857	up	0.017784324
IFIT1	1.9281499	up	0.009825654
IFN-OMEGA-4	1.2165959	up	0.031537097
IL10RA	1.3109044	up	0.007618589
IL13RA2	1.2234095	up	0.031530123
INPP1	1.6121705	up	0.04539076
IPO11	1.6942434	up	0.028802415
IRG1	1.2988328	up	0.018733868
ITIH1	1.4173031	up	0.043455612
JUN	1.2639325	up	0.033076253
KANK1	1.6061383	up	0.019916829
KPNA2	1.1473556	up	0.035920013
LATS2	1.4113225	up	0.010669898
LDB3	1.1897832	up	0.018393423
LDB3	1.6466472	up	0.033059057
LDLR	1.747672	up	0.0052309483
LDLR	1.7888163	up	0.0021007329
LDLR	1.9295712	up	0.042822417
LGI2	1.4213907	up	0.016707547
LGMN	1.236692	up	0.041313212
LGMN	1.2398609	up	0.03187108
LOC100049690	1.3252041	up	0.033960953
LOC100152091	1.3953458	up	0.04233427
LOC100152155	1.786298	up	0.0035112256
LOC100152494	1.7875584	up	0.016894916
LOC100152503	1.4372274	up	0.012710974
LOC100152590	1.8435212	up	0.03425818
LOC100152698	1.4366027	up	0.042095613
LOC100152734	1.8119309	up	0.04523958
LOC100153402	1.8520577	up	0.04656724
LOC100154167	1.347607	up	0.027479114

LOC100154715	1.2177752	up	0.04156987
LOC100155352	1.2882614	up	0.02955104
LOC100155492	1.7262831	up	0.03742717
LOC100156110	1.8008785	up	0.042840622
LOC100156161	1.1840732	up	0.033966053
LOC100156182	1.7520587	up	0.03106812
LOC100156419	1.4910651	up	0.000013089607
LOC100156419	1.8152783	up	0.0019221244
LOC100157270	1.969128	up	0.043427143
LOC100157783	2.6002529	up	0.014372887
LOC100157936	1.1879766	up	0.007225082
LOC100157957	1.5232888	up	0.047998723
LOC100157985	1.489193	up	0.027006004
LOC100270682	1.2794923	up	0.044041045
LOC100302368	1.7869091	up	0.022582406
LOC100512899	2.4196515	up	0.013015424
LOC100515779	2.5646083	up	0.02958339
LOC100515792	2.0068574	up	0.009346048
LOC100516366	1.6197059	up	0.02619869
LOC100517236	1.4605836	up	0.045615252
LOC100517350	1.2756352	up	0.03462723
LOC100517442	1.6959786	up	0.04375452
LOC100517869	1.2543691	up	0.045713656
LOC100518245	1.8155146	up	0.007874225
LOC100518251	1.391305	up	0.006147043
LOC100518294	1.1806058	up	0.013208308
LOC100518506	1.3667854	up	0.044032786
LOC100518609	1.1860012	up	0.003474073
LOC100519968	2.9755697	up	0.010597124
LOC100520046	1.0878243	up	0.0075571775
LOC100520074	1.2321769	up	0.02472799
LOC100521007	1.1137241	up	0.011016704
LOC100521680	1.2543179	up	0.028607098
LOC100523311	1.4152285	up	0.03990527
LOC100523628	1.5287662	up	0.006891712
LOC100523859	1.8797392	up	0.021364482
LOC100525259	1.5760227	up	0.031331107
LOC100525320	1.2135174	up	0.0434153
LOC100526249	1.0782926	up	0.024980342
LOC100620139	1.4682529	up	0.0020407417
LOC100621032	1.1667598	up	0.033984672
LOC100624543	1.4300469	up	0.04021251
LOC100626748	1.6900964	up	0.011504055
LOC100736918	1.4342036	up	0.037255086
LOC100737027	1.1524041	up	0.017863115
LOC100737557	2.1852124	up	0.024486827

LOC100738793	1.4156796	up	0.003912804
LOC102160253	2.1259966	up	0.007499105
LOC102161839	1.3030728	up	0.013127636
LOC396634	2.0708966	up	0.049912766
LOC641352	1.1719495	up	0.046551816
LOC733611	1.9121302	up	0.0370387
LOC733663	1.3315005	up	0.00991801
LOC767627	1.3543197	up	0.0049667526
LOXL1	1.1998348	up	0.022507813
M6PR	1.3012784	up	0.011884565
MED20	1.7045051	up	0.048002113
MGP	1.3384734	up	0.04959247
MGP	1.351236	up	0.02098766
MGRN1	1.5877984	up	0.04577321
MICAL1	1.6357198	up	0.033056833
MICU1	1.4009402	up	0.033278972
MIOS	1.8988636	up	0.014057496
MMP17	1.825496	up	0.0040620505
MST4	1.4738814	up	0.0043478236
MST4	1.8895844	up	0.04377174
MTERF	1.1329819	up	0.0183728
MUC13A	1.1173712	up	0.026450444
MUC13A	1.9795052	up	0.027073694
MYST4	1.159552	up	0.024150876
NCOA2	1.3774709	up	0.012367989
NCOR2	1.5742021	up	0.018908119
ND4	1.1679865	up	0.014489281
NKAIN3	1.2810637	up	0.03544915
NOS	1.1883782	up	0.017663052
NPTX1	1.5692022	up	0.036596026
NPY1R	1.6297002	up	0.048581287
OAT	1.768488	up	0.038734585
OLFML3	1.2950631	up	0.048176315
PANK1	1.5264459	up	0.024607379
PAPOLG	1.5378226	up	0.02969556
PAQR9	1.7633672	up	0.042305283
PCDH7	2.2949166	up	0.014122837
PDK1	2.5312395	up	0.013072117
PDLIM3	1.4034821	up	0.03874075
PEA15	1.4278253	up	0.01115184
PER2	1.5479078	up	0.026007887
PGK1	1.2680768	up	0.034177948
PGK1	1.3737106	up	0.03374703
PIK3CG	2.4548	up	0.019019544
PLCL1	1.7912076	up	0.025547037
PLEKHO1	1.5565647	up	0.028870033

PLEKHO1	1.6364363	up	0.014260619
PNAS-5	1.0843924	up	0.04985669
POFUT1	1.310855	up	0.01661808
POGZ	1.3759362	up	0.017217062
POLA1	1.2535543	up	0.02386375
POPDC2	1.6198289	up	0.034493364
PPA171	1.0973755	up	0.016245902
PPP2R1B	2.2778323	up	0.019539313
PRKRIP1	1.089529	up	0.0456449
PTGER3	2.4051373	up	0.03758311
PTGES	1.224353	up	0.040503453
PTPN6	1.8472255	up	0.021532182
PTPRE	1.6372497	up	0.026231794
RAB11A	1.1477039	up	0.030731577
RAB22A	1.7374029	up	0.018164327
RASA2	1.1211127	up	0.03170703
RASGEF1B	1.2571986	up	0.028505284
RBBP6	1.250169	up	0.0013311461
RFN19	1.3295047	up	0.013606617
RHOB	1.5147972	up	0.029227449
RHOB	1.5224067	up	0.013789866
RPL12	1.0938056	up	0.034664627
RRBP1	1.4627988	up	0.019566493
RTN4	1.3484373	up	0.043676857
RUNX1T1	1.2836969	up	0.048699427
SAV1	1.2917038	up	0.029646361
SCD	1.8390867	up	0.0432802
SEMA6D	1.3142364	up	0.03256538
SEPP1	1.3295012	up	0.018401593
SERPINA7	1.5055012	up	0.029117644
SGPP1	1.4016027	up	0.038358107
SHMT	1.2038836	up	0.030660193
SHPRH	1.6931089	up	0.038524278
SLA-1	1.7883658	up	0.0057109445
SLA-3	1.5458466	up	0.043258995
SLA-3	1.6546065	up	0.03965865
SLA-3	1.787371	up	0.00383546
SLA-5	1.4202198	up	0.030315625
SLC16A1	1.4898934	up	0.044815164
SLC16A1	1.5720029	up	0.035588242
SLC16A1	1.7043896	up	0.016590402
SLC1A3	1.3328676	up	0.011111339
SLC22A15	1.4638438	up	0.02067117
SLC25A6	1.2207767	up	0.031200726
SLC26A6	1.5420768	up	0.032840643
SLC29A1	1.6213374	up	0.04785215

SLC31A2	1.3903849	up	0.024145897
SLC3A1	1.6591324	up	0.012914752
SLC52A2	1.3194938	up	0.031131363
SLC7A9	2.396989	up	0.047750015
SLN	1.490621	up	0.020829206
SMG5	1.1381489	up	0.021617472
SMYD3	1.372421	up	0.004421303
SNCG	1.2536167	up	0.03502637
SNUPN	1.5811038	up	0.037026037
SOD1	1.2527089	up	0.00023244561
SOD1	1.31233	up	0.0046415934
SPARC	2.0247397	up	0.03231577
SPARC	2.2940772	up	0.043430258
SPARC	2.3436081	up	0.025674028
SPARCL1	1.2169908	up	0.045950104
SPATA22	1.6008383	up	0.012116949
SQLE	2.950152	up	0.023006437
SRP54	1.2393631	up	0.032337416
SRSF2	1.2435964	up	0.008161646
STAR	1.596258	up	0.046152614
STK17B	1.5650072	up	0.04272552
STOX2	1.3386644	up	0.04520017
SUPV3L1	2.2521648	up	0.039858606
SYNCRIP	1.3884069	up	0.02036687
TCF19	1.3669095	up	0.015003406
TH1L	1.9867269	up	0.015428733
THAP5	2.3285441	up	0.007623881
TMEM116	1.1455553	up	0.0010872785
TMEM170A	1.578228	up	0.024758425
TMEM70	1.1342896	up	0.031139813
TMEM8B	1.2236742	up	0.03251166
TMTC3	1.3625216	up	0.009552649
TNC	1.4185568	up	0.0091994535
TNFAIP1	1.1810068	up	0.032053396
TNIP1	1.3680582	up	0.022368299
TPM1	1.3918912	up	0.01285665
TPM1	1.4002101	up	0.017610548
TPM1	1.4221565	up	0.013644396
TPM1	1.4423454	up	0.0229019
TRAF3IP2	1.4696672	up	0.031727653
TRIM8	1.2770733	up	0.043689515
TRMU	1.8017379	up	0.0454378
TRPC1	1.3292438	up	0.0035140726
TTI1	1.4875463	up	0.0045166616
UBE2Q1	1.0701766	up	0.046863902
UPF3	1.6018484	up	0.035278574

VCP	1.1899145	up	0.0020858312
VPS36	1.3943307	up	0.011787788
VTN	1.4451907	up	0.031931285
WDR96	1.2083397	up	0.008243847
WEE1	1.533015	up	0.04697505
WNT10B	1.1923258	up	0.024994327
WNT10B	1.8207701	up	0.0071076755
WT1	1.6200063	up	0.048532404
YWHAB	1.3189137	up	0.016282333
YWHAB	1.3923551	up	0.02069697
ZBED5	1.3236853	up	0.016679404
ZC3H11A	1.8289568	up	0.024097836
ZIC2	1.1905708	up	0.022957966
ZNF639	1.1212027	up	0.032027975
AARS2	1.7721819	down	0.00421577
AASDH	2.0541966	down	0.032815814
ACSS1	1.3950557	down	0.0083460575
ADAMTS1	1.6531208	down	0.026713423
ADAP1	1.2315089	down	0.014293579
AKT3	1.2018864	down	0.0054142787
ALDH5A1	1.487971	down	0.0038318236
ANAPC7	1.2423421	down	0.01201133
ANPEP	2.6199598	down	0.042388365
ANXA9	1.1472259	down	0.015107064
ANXA9	1.2733428	down	0.039155636
AR	1.2029912	down	0.035917543
ARGLU1	1.6731913	down	0.012323383
ARHGAP28	1.1785779	down	0.013952512
ATP6V1A	1.1111553	down	0.033786062
ATP9A	1.3242505	down	0.039991185
ATRNL1	1.4207208	down	0.00006683815
AURKB	1.2833034	down	0.033545528
BAX	1.728904	down	0.0033640638
BET1L	1.5563059	down	0.039090585
BGN	1.1213146	down	0.00032314897
BIN1	1.2862455	down	0.01844637
BTG2	2.1198573	down	0.024137484
BTG3	1.3001375	down	0.04837784
C18H7orf58	1.5302438	down	0.013962582
C1H14orf101	1.0592332	down	0.03595831
C1H9orf156	1.4430345	down	0.03211644
C6	1.5627232	down	0.026255427
CACNG7	1.1493868	down	0.0090908045
CART	1.344743	down	0.02050304
CCDC85C	1.4980338	down	0.04206343
CD86	1.1924443	down	0.038280115

CDC40	1.8874643	down	0.017813725
CDC42BPB	1.2919327	down	0.032483872
CDK5RAP1	1.2208419	down	0.036893412
CDK5RAP2	1.6349742	down	0.004603899
CDKN1B	1.2448862	down	0.04702668
CEP85	1.6509668	down	0.039276205
CETN3	1.5986744	down	0.04910194
CHIA	1.4114048	down	0.023644319
CHSY1	1.4023576	down	0.01604909
CIDE-B	1.1559358	down	0.034960784
CLDN11	1.3258367	down	0.015658204
CLDN8	1.3157717	down	0.039494477
CNTN5	1.2871014	down	0.028541287
COQ3	1.1648089	down	0.04251775
COQ3	1.2570536	down	0.02832067
CTNNA2	1.9770147	down	0.018314244
CTSF	1.5199095	down	0.0417031
CYP39A1	1.1910385	down	0.0021178264
CYP3A39	2.7377567	down	0.03270455
DIO1	1.4061508	down	0.027412776
DLK1	1.1820922	down	0.034156542
DNAJB1	1.6270254	down	0.044511456
DNAJC14	1.7389983	down	0.011405737
DOK5	1.9217205	down	0.007574935
DSPP	1.7398368	down	0.022162098
EDN3	1.59416	down	0.007460998
EHF	1.2675034	down	0.044565223
ELOVL2	1.3606147	down	0.029462788
ENTPD7	1.6604615	down	0.028627595
EPCAM	1.409533	down	0.015865486
EPCAM	3.3074265	down	0.034928028
EPCAM	3.4634197	down	0.043330666
EPHA7	1.2310827	down	0.025917592
EPS15L1	1.241959	down	0.03178049
EPYC	1.485424	down	0.016289119
ERMARD	2.0363538	down	0.018768657
EVI5	1.1254203	down	0.04308568
F2	1.3628912	down	0.0043250076
FAIM	1.8450829	down	0.030077063
FBXO7	1.1909825	down	0.031963333
FEM1B	1.649812	down	0.008362856
FGF9	1.7021793	down	0.021417066
FMO1	1.1598971	down	0.0073210294
FST	1.5687408	down	0.034405828
FST	2.1544855	down	0.028576152
GADD45A	2.0218768	down	0.047872405

GADD45G	2.17673	down	0.032438044
GALP	1.8037207	down	0.044506844
GAPVD1	1.4621634	down	0.037288036
GATC	1.3163283	down	0.04142384
GGH	1.4222107	down	0.007688622
GLCCI1	1.4006737	down	0.010714654
GLRX	1.4213327	down	0.046515588
GLRX	1.4504206	down	0.040492047
GLT8D1	1.0291268	down	0.004946954
GLUD1	2.0338306	down	0.034725428
GOLPH3L	1.2773547	down	0.038275834
GPR56	1.8911479	down	0.041843507
GSDMA	3.9570465	down	0.014480304
GTF2B	1.141925	down	0.020752197
HAT	1.6751764	down	0.049719308
HBB	3.9340596	down	0.013436006
HLTF	1.5486664	down	0.018195914
HMOX1	1.625311	down	0.0419278
HNF4A	1.8415256	down	0.023498327
HOMER2	1.3177134	down	0.041649353
HSPCB	1.2795125	down	0.04711462
ID2	1.6244256	down	0.043551
ID3	1.9166529	down	0.008188853
IL24	1.3496896	down	0.026575124
INA	1.5979757	down	0.0026434488
ISG20	1.5348516	down	0.049666718
ITGB3	1.4804847	down	0.030216986
KCND2	1.2523469	down	0.00075914955
KCNQ5	1.4724151	down	0.031513315
KHDRBS3	2.0650403	down	0.0042364774
KHDRBS3	2.1012535	down	0.015361872
KHDRBS3	2.1426203	down	0.017743843
KHDRBS3	2.2834094	down	0.01730603
KHDRBS3	2.3211899	down	0.012825805
KIAA1671	1.1659051	down	0.040603157
KLF10	1.9138396	down	0.028874954
KLF10	2.2685494	down	0.00017082757
KLF3	1.3569616	down	0.04490282
KPNA7	1.5883642	down	0.033127576
KRT2	1.260256	down	0.019494144
LBX1	1.3959422	down	0.04799423
LGALS12	1.499131	down	0.024557544
LGALS3	1.2456771	down	0.028585529
LGALS3	2.0333872	down	0.0421622
LGALS3	2.1981158	down	0.017045425
LGALS3	2.216682	down	0.016149474

LGALS3	2.222093	down	0.01888185
LGALS3	2.2234333	down	0.01928116
LGALS3	2.2732856	down	0.024324043
LGALS3	2.4416099	down	0.046687577
LGALS4	1.1295307	down	0.005289933
LHFPL1	2.2788618	down	0.020030169
LOC100049695	1.1405556	down	0.0354861
LOC100151929	1.3989666	down	0.015253093
LOC100152291	1.2618501	down	0.023331122
LOC100152299	1.3151187	down	0.049687088
LOC100152565	1.4764016	down	0.0029627427
LOC100152612	1.3019174	down	0.017586842
LOC100152729	1.921436	down	0.04972795
LOC100153269	1.3672438	down	0.039456658
LOC100153615	1.8232172	down	0.010603847
LOC100153787	1.5614918	down	0.02602327
LOC100154770	1.8792025	down	0.044351827
LOC100155159	1.3301821	down	0.024903873
LOC100155688	1.4923105	down	0.04950214
LOC100156584	1.3907703	down	0.012540668
LOC100156639	1.1698071	down	0.0036618665
LOC100156887	1.481126	down	0.03594088
LOC100157434	1.8219538	down	0.0011767258
LOC100157925	2.5213811	down	0.047997758
LOC100169745	1.6329405	down	0.016730724
LOC100169745	2.22716	down	0.007695988
LOC100511354	1.5433291	down	0.032765586
LOC100511782	1.4081659	down	0.0112582445
LOC100512253	1.2044806	down	0.025238594
LOC100512509	1.3577305	down	0.002332527
LOC100512562	1.8717107	down	0.032203227
LOC100513149	1.3024981	down	0.012614323
LOC100515931	1.8534007	down	0.007474691
LOC100515931	2.0535665	down	0.0071202465
LOC100518983	1.2625031	down	0.033770785
LOC100519123	1.211838	down	0.005399011
LOC100520599	1.1932292	down	0.030947752
LOC100524508	1.6722105	down	0.0192184
LOC100526102	2.5666537	down	0.03527564
LOC100620398	1.7300034	down	0.0069321366
LOC100620666	1.2476581	down	0.040192783
LOC100620819	3.2024033	down	0.026737232
LOC100620995	1.1781998	down	0.039735615
LOC100621284	1.2800016	down	0.04544873
LOC100623462	1.3064005	down	0.044951532
LOC100624417	1.2539635	down	0.023779126

LOC100628150	1.2315379	down	0.049514066
LOC100628150	1.3236637	down	0.025465949
LOC100736775	1.5234376	down	0.0007965671
LOC100736806	1.9941275	down	0.013430804
LOC100736874	1.8609222	down	0.020313729
LOC100737266	1.4050204	down	0.027816996
LOC100737768	2.4258935	down	0.0015828351
LOC100739033	1.6343396	down	0.0123428255
LOC102162243	1.2351146	down	0.04577952
LOC102163547	1.5348383	down	0.0490211
LOC102163574	1.4724588	down	0.04554768
LOC102163734	1.2160215	down	0.018294916
LOC102163861	1.2189016	down	0.043579888
LOC102164781	1.3393023	down	0.034192156
LOC102164954	1.2370113	down	0.02722218
LOC102167917	1.5340241	down	0.00036699264
LOC396679	1.2813538	down	0.033839162
LOC733605	1.0933511	down	0.04760061
LOC780431	1.3289349	down	0.034953795
LPIN1	1.4267651	down	0.042252485
LRRN3	1.4362037	down	0.01995135
LY9	1.6254255	down	0.0028852976
MCM3AP	1.3897972	down	0.010057979
MCTP2	1.1906788	down	0.0024719408
MGAT4C	1.5096562	down	0.0012958671
MMP11	1.6546843	down	0.008278942
MMP16	1.1888505	down	0.04363636
MRAP2	1.1204658	down	0.015767938
MTTP	1.4641343	down	0.010796297
MYOT	1.4410251	down	0.007026133
MYOT	2.2101817	down	0.03740839
MYOT	2.7280097	down	0.0089283725
NBR1	1.4957447	down	0.041660193
NCOA1	1.1195726	down	0.048318878
NEB	1.3810693	down	0.023045968
NHEJ1	1.2790036	down	0.037702095
NMB	1.3421619	down	0.037437465
NMB	1.3894237	down	0.014297419
NOR-1	4.862286	down	0.04666066
NOR-1	5.3672457	down	0.038859688
NOVA1	1.2945522	down	0.01252614
NPHS2	1.2045047	down	0.025008466
NR3C1	1.5135373	down	0.008404306
NR4A2	1.7046522	down	0.04775998
NSMCE4A	1.0279634	down	0.0246208
NUP188	1.4309392	down	0.04019897

NYX	1.5136353	down	0.045229886
OAZ3	1.2101022	down	0.025951829
P2RY2	1.3741019	down	0.04723842
PALMD	1.5311724	down	0.011830388
PDE4B	2.1586864	down	0.024091905
PGCP	1.2751501	down	0.029302636
PIGO	1.3340145	down	0.011440599
PION	1.8956909	down	0.0365023
PKC	1.4256747	down	0.0041887583
PKD2L1	1.6156584	down	0.018420087
PLAT	1.6727663	down	0.043046348
PLCD4	2.9750826	down	0.03054151
PMM1	1.3914207	down	0.0065304213
PNLIPRP1	1.2062948	down	0.040298767
POLL	1.5219187	down	0.028515875
POLR3GL	1.1392826	down	0.033035945
PON1	1.2640402	down	0.035218254
PPAG3	1.1981068	down	0.027250614
PPP1R12C	3.9043336	down	0.0019551248
PPP3CA	1.4683805	down	0.019145627
PRDX4	1.3067784	down	0.03206749
PRPF31	1.0865598	down	0.046719488
PRPF8	1.2582291	down	0.0025728426
PSP-I	1.4769206	down	0.039442535
PTGFR	1.0961545	down	0.0085731875
PTPN21	1.5028298	down	0.01437755
RAB20	1.3744552	down	0.018166449
RAD18	1.2665807	down	0.0014491855
RAE1	1.2085986	down	0.031356584
RAP1A	1.5065484	down	0.047685068
RASGRP3	1.4965605	down	0.049094427
RBM12	1.2915263	down	0.03917315
RBM26	1.8190947	down	0.0008807816
RBM38	1.4221172	down	0.021001723
RFC4	1.6971322	down	0.0410653
RING1	1.1023014	down	0.020143205
RN18S	1.6077378	down	0.031585135
RNF144B	1.1672062	down	0.010817245
RPA39	1.1487651	down	0.011788881
RPL10A	1.1882504	down	0.046082526
RPL14	1.1059635	down	0.009875451
RPL14	1.1995071	down	0.049331516
RPL14	1.2549564	down	0.03783008
RPL18	1.2394418	down	0.006794939
RPL18	1.3457848	down	0.0028783204
RPL29	1.4044158	down	0.008002348

RPL7	2.5449276	down	0.00059197436
RPLP1	1.2712915	down	0.041211035
RPLP1	1.3233528	down	0.012269579
RPRD2	1.7568525	down	0.010733489
RPS12	1.2821274	down	0.03902218
RPS21	1.2549045	down	0.023834862
RPS21	1.2757963	down	0.02059349
RPS21	1.296567	down	0.015765637
RUNDC3A	1.48132	down	0.047810968
SAL1	1.3235818	down	0.031487025
SBNO1	2.790988	down	0.032951936
SDC2	1.4150164	down	0.033524826
SEC23B	1.1509472	down	0.0129893385
SELK	1.1953145	down	0.014825538
SELK	1.2079155	down	0.02191504
SERBP1	1.6663343	down	0.0033083765
SERPINB7	1.3297417	down	0.040665574
SERTM1	1.7138011	down	0.042059712
SGPP1	1.299596	down	0.036580425
SH3D19	1.7633697	down	0.015640631
SIRPA	2.141955	down	0.0074844556
SLC10A7	1.7965424	down	0.0016544234
SLC25A2	1.5279303	down	0.047676947
SLC5A5	1.1641595	down	0.030013422
SLC7A7	1.2149588	down	0.0034888778
SLK	1.6037107	down	0.034038953
SLK	1.60589	down	0.010021771
SMG5	1.1523811	down	0.004386738
SMPD4	1.1266834	down	0.015893256
SPCS1	1.1967579	down	0.024351101
SPDYA	1.4293532	down	0.019802704
SPIB	1.5354944	down	0.0025394112
SPMI	1.5604485	down	0.020856282
SQLE	1.4975674	down	0.028630067
ST3GAL1	1.7667004	down	0.034727205
STAT4	1.4687356	down	0.045409236
STC1	2.0778716	down	0.006214476
SYNGR1	1.3175001	down	0.040250793
SYNM	1.3493866	down	0.036676608
TAS1R3	1.4202038	down	0.02584394
TATDN1	1.3485711	down	0.040854387
TBC1D10A	1.7338972	down	0.017037174
TBC1D10A	1.7352567	down	0.03916579
TBP10	1.161113	down	0.03760036
TBR1	1.2638421	down	0.031028204
TBX2	1.4932145	down	0.03308507

TCTE1	1.4090763	down	0.00013007181
TGS1	1.2816865	down	0.04329383
TLR9	1.2252517	down	0.044655867
TMEM63B	1.463143	down	0.025951441
TMEM70	1.2774985	down	0.048402213
TMEM8B	1.4401746	down	0.04353159
TOP1	1.2940892	down	0.042268135
TRAF5	1.3157303	down	0.034243237
TRAP1	1.4398468	down	0.017740559
TRAV41	1.496399	down	0.028905924
TRPM7	1.1765898	down	0.047938224
TRPV1	1.8254371	down	0.0132454205
TRUB2	1.2771617	down	0.017126214
TSPAN14	1.707419	down	0.016439157
TTC13	1.1894366	down	0.027554667
TTI1	1.2061981	down	0.008603581
UBE2E2	1.4657907	down	0.038308535
UBIE	1.2273344	down	0.026260791
UBL5	1.2222928	down	0.011895648
USP11	1.2360735	down	0.010778685
VPS36	1.4921602	down	0.034574635
WASL	1.2381734	down	0.01983351
WDR11	1.219518	down	0.014020555
WDR3	1.5155419	down	0.03926753
WDR3	1.9234115	down	0.049103998
WNT9A	1.2245679	down	0.009137924
XPO5	1.1747596	down	0.049170244
YIPF3	1.219979	down	0.036159273
YIPF3	1.2267056	down	0.023434056
YIPF5	1.341141	down	0.04435715
ZBTB2	1.323764	down	0.03790329
ZFP36L1	1.452387	down	0.036316086
ZFP36L1	1.5339545	down	0.046684578
ZNF750	1.6494609	down	0.044737726
