# Supplementary material for: Transcriptomic analysis of the myometrium during peri-implantation period and luteolysis–the study on the pig model
Source: Funct Integr Genomics. 2014 Sep 21;14(4):673–82. doi: 10.1007/s10142-014-0401-4 (PMC4233113; doi:10.1007/s10142-014-0401-4)
Supplement: Supplementary file 2 — (PDF 27 kb) [file 10142_2014_401_MOESM2_ESM.pdf]

Article title: Transcriptomic analyses of the myometrium during periimplantation period and luteolysis - the study on the pig model

Journal name: Functional & Integrative Genomics

Author names: Anita Franczak*, Bartosz Wojciechowicz, Justyna Kolakowska, Kamila Zglejc, Genowefa Kotwica

Affiliation: Department of Animal Physiology, Faculty of Biology and Biotechnology, Oczapowski 1A, University of Warmia and Mazury in Olsztyn, 10-718 Olsztyn, Poland

*Corresponding author: phone: +48 89 5233201; fax: +48 89 5233937
E-mail address: anitaf@uwm.edu.pl (Anita Franczak)

Supplementary Table 2. Top twenty up- and downregulated genes in the myometrium

No.	Gene name	Fold-change	Regulation during pregnancy	P-value
1.	*ETV3*	4.66	up	0.033
2.	*ENPP1*	4.53	up	0.024
3.	*CCR1*	3.49	up	0.001
4.	*CCR3*	3.49	up	0.001
5.	*CCXCR1*	3.49	up	0.001
6.	*FYCO1*	3.49	up	0.001
7.	*IARS2*	3.09	up	0.010
8.	*LOC100519968*	2.98	up	0.010
9.	*SQLE*	2.95	up	0.023
10.	*HGFR*	2.80	up	0.015
11.	*CA3*	2.65	up	0.011
12.	*ARMC9*	2.63	up	0.035
13.	*LOC100157783*	2.60	up	0.014
14.	*LOC100515779*	2.56	up	0.030
15.	*HOXA13*	2.54	up	0.048
16.	*PDK1*	2.53	up	0.013
17.	*PIK3CG*	2.45	up	0.019
18.	*ASPN*	2.43	up	0.004
19.	*LOC100512899*	2.42	up	0.013
20.	*PTGER3*	2.40	up	0.038
1.	*NOR-1*	5.37	down	0.039
2.	*NOR-1*	4.86	down	0.047
3.	*GSDMA*	3.96	down	0.014
4.	*HBB*	3.93	down	0.013
5.	*PPP1R12C*	3.90	down	0.002
6.	*EPCAM*	3.46	down	0.043
7.	*EPCAM*	3.30	down	0.035
8.	*LOC100620819*	3.20	down	0.027
9.	*PLCD4*	2.97	down	0.030
10.	*SBNO1*	2.79	down	0.033
11.	*CYP3A39*	2.74	down	0.033
12.	*MYOT*	2.73	down	0.009

13.	*ANPEP*	2.62	down	0.042
14.	*LOC100526102*	2.57	down	0.035
15.	*RPL7*	2.54	down	0.001
16.	*LOC100157925*	2.52	down	0.048
17.	*LGALS3*	2.44	down	0.047
18.	*LOC100737768*	2.43	down	0.001
19.	*KHDRBS3*	2.32	down	0.013
20.	*KHDRBS3*	2.28	down	0.017
